# Natural forests of the world – a 2020 baseline for deforestation and degradation monitoring

**DOI:** 10.1038/s41597-025-06097-z

**Published:** 2025-11-13

**Authors:** Maxim Neumann, Anton Raichuk, Yuchang Jiang, Mélanie Rey, Radost Stanimirova, Michelle J. Sims, Sarah Carter, Elizabeth Goldman, Keith Anderson, Petra Poklukar, Katelyn Tarrio, Myroslava Lesiv, Steffen Fritz, Nicholas Clinton, Charlotte Stanton, Dan Morris, Drew Purves

**Affiliations:** 1 Google DeepMind, Zurich, Switzerland; 2 World Resources Institute, Washington DC, USA; 3 University of Zurich, Zurich, Switzerland; 4 Google Research, Mountain View, CA USA; 5 Google, Mountain View, CA USA; 6 International Institute for Applied Systems Analysis, Laxenburg, Austria

**Keywords:** Forest ecology, Environmental impact

## Abstract

Informed decisions to reduce deforestation, protect biodiversity, and curb carbon emissions require not just knowing where forests are, but understanding their composition. Identifying natural forests, which serve as critical biodiversity hotspots and major carbon sinks, is particularly valuable. We developed a novel global natural forest map for 2020 at 10 m resolution. This map can support initiatives like the European Union’s Deforestation Regulation (EUDR) and other forest monitoring or conservation efforts that require a comprehensive baseline for monitoring deforestation and degradation. The globally consistent map represents the probability of natural forest presence, enabling nuanced analysis and regional adaptation for decision-making. Evaluation using a global independent validation dataset demonstrated an overall accuracy of about 92%.

## Background & Summary

Forests are critical assets in global efforts to mitigate climate change, conserve biodiversity and support livelihoods. They help stabilize the global climate by absorbing significant amounts of greenhouse gases^1^. Forest ecosystems harbor over 80% of the world’s threatened species, making them essential for biodiversity conservation^2^. Additionally, forests support the livelihoods of over 1.6 billion people worldwide, including nearly 70 million Indigenous Peoples, by providing food, shelter, medicine and economic opportunities^3,4^. While the importance of forests is global, the ecological roles and disturbance regimes of tropical, temperate and boreal forests can differ substantially, influencing how loss, degradation, biodiversity maintenance, and carbon changes occur across forest climate domains. Despite the critical role that forests play, deforestation continues at an alarming rate^5^ primarily driven by the expansion of agricultural land^6^. In response, more than 140 countries have pledged to end forest loss by 2030, and numerous voluntary and regulatory initiatives have emerged to reduce the impact of agriculture on forests^7^. These include corporate zero-deforestation commitments and policies such as the European Union Deforestation Regulation (EUDR), which aims to ensure that products imported into the EU market (e.g., cocoa, coffee, oil palm, rubber, cattle, soy) do not come from areas that were deforested or degraded after December 31, 2020^8^. Monitoring and achieving these goals requires accurate and comprehensive depictions of global natural forest cover, accounting for the distinct ecological characteristics and disturbance regimes of tropical, temperate, and boreal biomes.

A number of datasets map tree cover globally for various time periods^9,10^, including as a class within land cover datasets^11–14^. However, these datasets are a biophysical measure of woody vegetation often based on height or canopy density and do not distinguish natural forests – such as primary forests and naturally regenerating forests – from planted trees, including tree crops, wood fiber plantations, or agroforestry systems. When such datasets are used for forest monitoring, changes within planted forests, such as harvesting, felling of older agricultural trees, and loss of other non-natural tree cover are often conflated with deforestation of natural forests, complicating data interpretation and potentially leading to wasted investigatory resources. Available data that distinguishes forest types, such as natural or planted forests, are more limited; for example, Vancutsem *et al*.^15^ separate plantations from undisturbed and degraded forests, but limit their study area to moist forest in the tropics, while Lesiv *et al*.^16^ map forest management types globally, but only for the year 2015 and at 100 m resolution. Datasets that explicitly consider disturbance regimes specific to tropical, temperate or boreal climate domains remain scarce. More recently, a number of global forest maps have been developed for the year 2020 by combining multiple datasets to meet specific definitions for various intended applications, such as compliance with EUDR^8,17– 20^, corporate target-setting with the Science Based Targets Network (SBTN)^21^, and Intergovernmental Panel on Climate Change (IPCC) forest biomass estimates^22,23^. However, because these maps were created by combining various input datasets, they are subject to a number of limitations, including inconsistent quality in certain geographic regions or for specific forest types due to limitations of available input data^18,19,21,23^. Furthermore, the ability to update these maps in the future is contingent upon updates to the input data.

This study fills an important data gap by moving beyond tree cover to provide a natural forest map for 2020 that can be used as a baseline for forest monitoring. Under EUDR, which requires companies to provide the geographic coordinates of sourcing areas and assessment of deforestation or degradation risk for these locations, this data can support companies in conducting due diligence by providing a baseline companies can use to evaluate if commodities were produced in areas that have been deforested or degraded after 2020. Furthermore, this data can support forest monitoring efforts more broadly by providing a baseline that can be adopted across tropical, temperate and boreal forests by distinguishing between natural forest loss versus rotations or harvest of tree plantations or tree crops. This critical advancement supports forest conservation and sustainable management efforts, as well progress toward global climate and biodiversity goals.

**Fig. 1:**
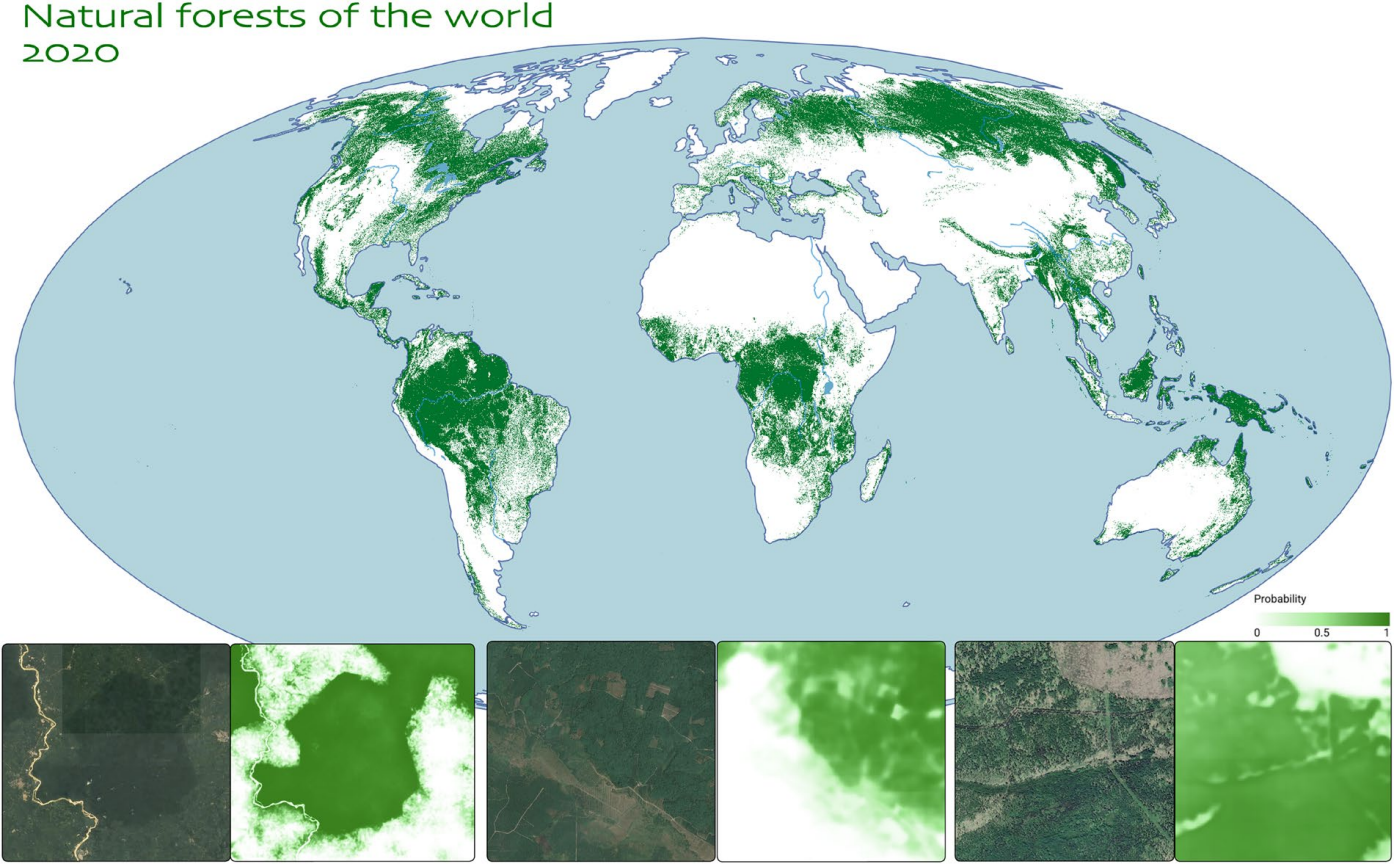
The global extent of natural forests in 2020 (according to our model, and based on the probability threshold of 0.52) with zoom-in examples (from left to right: Amazon Basin in Brazil, deforestation frontier in Indonesia, and boreal forest in Western Canada).

The main objective of this paper is the generation of a novel, globally consistent, calibrated, probabilistic mapping of the natural forests of the world (NFW). We trained a single model for the entire world at 10 m resolution. We performed a large-scale (about 2 million square kilometers (2M km^2^)) global stratified sampling of land cover across the globe for the training data, from a global sample of 1.2 million non-overlapping locations, so that the model saw all possible land cover types, could distinguish coarse categories, and had the capability to discriminate natural forest from other tree cover (planted forest, tree crops, etc.) and non-forest environments (Table 1). We constructed the training labels from diverse sources, including manually labeled high-quality annotations as well as weakly labeled inference results. We trained a novel multi-modal, multi-temporal transformer neural network model on satellite remote sensing data (Sentinel-2^24^) at 10 m resolution. It performed semantic segmentation taking local spatial context as well as seasonal temporal variation into account. In addition to multi-spectral inputs, the model used topography information as well as geographic location information. We performed inference on the trained model to generate a global, consistent map of natural forest at 10 m resolution for the year 2020. We calibrated the predicted pseudo-probabilities of the natural forest class to better represent the actual probability of a given pixel being a natural forest. Providing these probabilities rather than a fixed binary classification allows users to adapt the natural forest prediction to a specific climate domain or to the regional context and user application goals. We evaluated the generated map on a validation dataset based on the Global Forest Management stratified validation dataset^16^ updated for the year 2020 (Fig. 1).

**Table 1:** Forest definitions used in this study.

Land type	Definition
forest	land area with more than 0.5 hectares, with trees higher than 5 meters and canopy cover greater than 10%. it includes natural and planted forests and excludes everything else (in particular other land with tree cover that doesn’t meet the definitions above or is predominantly used for agriculture (tree crops) or other land use).
Natural forest	Undisturbed forest where no major human impacts have been detected via satellite imagery in recent history (since the year 1984); naturally regenerating secondary forests; and managed natural forests with no signs of planting. Managed natural forests may be subject to logging, harvesting of forest products, or other low-intensity activities that do not substantially alter forest structure, so long as clear signs of planting have not been detected. This category also includes degraded forests (so long as they have not been converted to a non-forest land use, and degradation does not result in the sustained reduction of tree cover below the height and tree canopy thresholds). Mangroves and savannas are included if they fulfill the forest and naturalness definitions above.
Planted forest	Stands of planted trees, other than tree crops, with visible signs of planting, such as rows and/or even age distribution. Typically grown for wood and wood fiber production or as ecosystem protection against wind and/or soil erosion.
Tree crops	Perennial trees that produce agricultural products, such as rubber, oil palm, coffee, cocoa, and orchards.
Other land cover types	Other vegetation (including agriculture, as well as savannas and urban trees that do not fulfill the definitions above), human built environments, water bodies, permanent ice/snow, and bare/sparse vegetation land covers.

## Methods

**Fig. 2:**
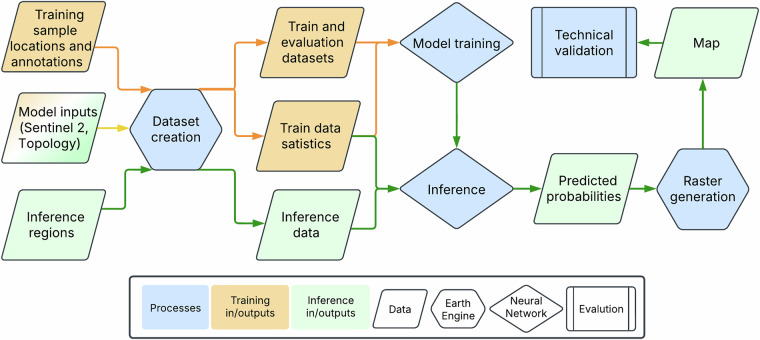
Study design and the overall flow of data for model training, global map construction and the final technical validation.

Our approach harmonized multiple labeled data sources to train a global deep learning semantic segmentation model for estimating the probability of natural forest. This model exploits spectral, temporal, and textural information from satellite remote sensing. For reference, Fig. 2 provides a diagram of study design and overall data flow for model training, evaluation, and final map generation.

### Definitions

The Food and Agriculture Organization of the United Nations (FAO) offers a widely used definition of forests: “Land spanning more than 0.5 hectares with trees higher than 5 meters and a canopy cover of more than 10 percent, or trees able to reach these thresholds in situ. It does not include land that is predominantly under agricultural or urban land use”^25,26^. The FAO goes on to define “Naturally regenerating forest” as “Forest predominantly composed of trees established through natural regeneration”. This includes several explanatory notes: 1. Includes forests for which it is not possible to distinguish whether planted or naturally regenerated. 2. Includes forests with a mix of naturally regenerated native tree species and planted/seeded trees, and where the naturally regenerated trees are expected to constitute the major part of the growing stock at stand maturity. 3. Includes coppice from trees originally established through natural regeneration. 4. Includes naturally regenerated trees of introduced species. However, some aspects of these definitions cannot be mapped using earth observation data alone, such as “trees able to reach these threshold in situ.” Therefore, we adapted our natural forest definition to one which can be used in a remote sensing application. In our study, natural forests include primary forests, naturally regenerating secondary forests, managed natural forests, and degraded forests that have not been converted to another use. Table 1 summarizes the category definitions we used to map natural forest in this study.

### Training data creation

Training a deep learning model to recognize natural forest at 10 m resolution requires numerous high-quality training examples. We first sampled *positive* samples containing natural forests (class 1), and then included supplementary classes of *negative* samples. We divided the negatives into *hard negatives*—land cover classes visually similar to natural forests in satellite imagery, including planted forests (class 2), tree crop plantations (class 3) and some other vegetation (class 4)—and *soft negatives*—more distinct land cover classes—including human built environments (class 5), water bodies (class 6), permanent ice and snow (class 7), as well as bare ground or sparse vegetation (class 8). We found it beneficial for the model to learn these classes separately to develop a nuanced understanding of land cover types; a simpler binary segmentation (natural forest vs. other) did not perform as well.

In the first stage (“locations sampling”), we constructed a global sample of 1.2 million non-overlapping locations, each covering 1280  × 1280 m^2^ area (totaling approx. 2 million square kilometers). We initially prioritized locations with known natural forest and other tree cover (*positives* and *hard negatives*), incorporating samples where ground truth information (manual/in-situ labels) for the forest types was available (Table 2). Additionally, we sampled random locations within every 100  × 100 km^2^ region containing land globally to include other land cover types and underrepresented areas.

**Table 2:** Label sources for constructing labels for model training. The class column denotes for which classes the source was used (1: natural forest, 2: planted forest, 3: tree crops, 4: other vegetation, 5: built environments, 6: water, 7: ice and snow, and 8: bare or very sparse vegetation).

Name	Classes	Type	Description
PHTF	1	R,I	Primary humid tropical forest (PHTF) for the year 2001^50^ at 30 m resolution.
Boreal	1	R,I	Forest age (FA) in the boreal forest biome^51^ is used to identify primary and old secondary forest stands older than 20 years in 2020 at 30 m resolution.
European Primary	1	V,C	European primary forest database (v2)^52^ harmonizing 48 different datasets in the form of polygons and points verified by Landsat time series.
Canada Primary	1	R,I	Estimated forest age in Canada based on Landsat temporal composites and allometric equations coupled with forest structure and productivity metrics^53^, that we threshold at 50 years to obtain a conservative range of primary forests.
USA MOG	1	R,I	Mature and old-growth (MOG) forests over the contiguous United States^54^ at 30 m resolution, that we threshold at a minimum index of 7 (in the range 1 to 10) to include mature naturally regenerating forests.
GFT2020	1-2	R,C	JRC global map of forest types (FT) at 10 m spatial resolution^19^. Classes 1 and 10 are used as for natural forest, while class 20 is used for planted forest labels.
TMF	1-2	R,I	JRC tropical moist forest (TMF) types^15^. Classes 10, 11, 12, 51, 52, 53, 54, 55, 56 as well as 21, 22, 23, 24, 25, 26, 31, 32, 33, 63 are mapped to natural forest labels, while classes 92 and 93 are used for planted forest labels.
SDPT (v2)	2-3	V,C	The Spatial Database of Planted Trees (SDPT) dataset contains a set of planted forest and tree crops polygons^55,56^.
ETH cocoa	3	R,I	Probability of cocoa growing area at 10 m resolution^57^, that we binarize at probability threshold of 0.9.
CORINE	3	R,I	Copernicus CORINE land cover map over Europe^58^.
CDL	3	R,I	USDA’s Cropland Data Layers (CDL) of the United States^59^.
Tree crops	3	V,M	A combination of tree crop commodities in the form of polygons (or squares around points) from the various public sources^60–72^.
WorldCover	4-8	R	ESA’s 10 m WorldCover land cover land use classification (including classes for built, snow/ice, bare, and water)^12^.
SBTN	1-2, 4-8	R,C	Natural land map from the Science Based Targets Network (SBTN)^21^ at 30 m resolution.

The type column denotes whether the data is a rasterized map (R) or vector data (polygons, points) (V), and whether the source involved manual inspection (M), model inference (I), or a combination (C).

**Fig. 3:**
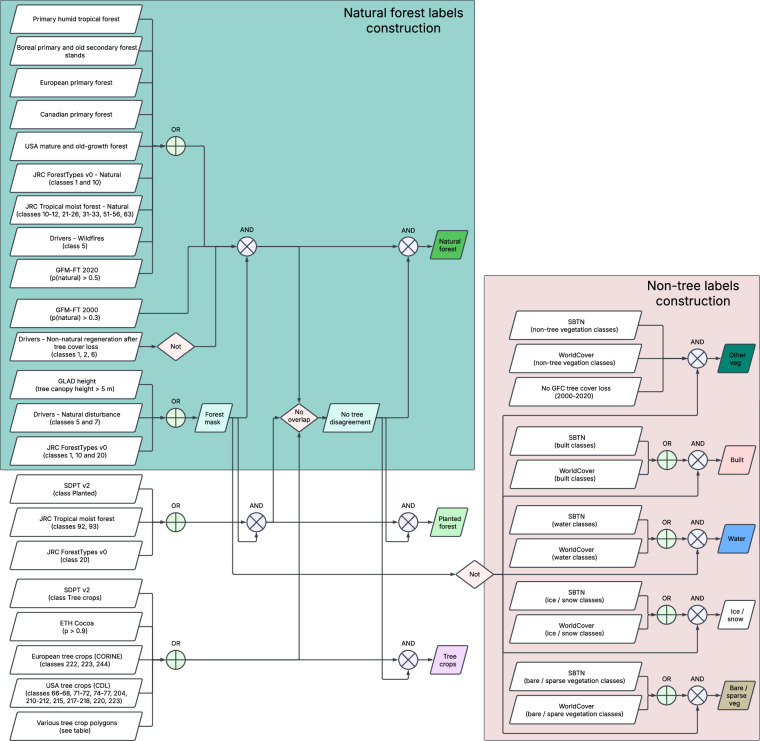
Diagram of label assignment based on label data sources.

In the next stage (“class assignment”), we assigned one of eight labels (and an extra “unknown” label, class 0) to each 10 m pixel within each sample location (there are 128^2^ = 16,384 pixels per sample). We used the label construction process as outlined in Fig. 3, based on the data sources described in Table 2 and Table 3. We designated areas as unknown (class 0) where data sources disagreed on a label, or where no label candidate existed. We aimed to make the best use of all available datasets to create labels for model training. Among others, we included the JRC Forest Types v0^19^ as one of the sources, in addition to our retrained GFM-FT 2020 map based on updated GFM 2020 training data (an update to^16^). Some assigned labels could be spurious, especially if coming from other weaker machine learning model inferences; however, we expected the model could learn to identify and potentially reclassify these label errors. The decisions for the labels construction algorithm (Fig. 3) were data-driven; we iterated across many different label sources and combination configurations before arriving at them. The final presented version optimized model training and map quality, based on evaluation results and external reviewers feedback.

**Table 3:** Supporting layers for constructing labels for model training.

Name	Description
GLAD GFC	Global Forest Change (GFC) data contains global layers of tree cover, forest gain and loss, with the year of forest loss, along with Landsat 7 cloud-free composite^9^. We used the GFC tree cover (GFC TC) layer for the year 2000, and the forest loss year layer (between 2000 and 2020) to create masks for tree cover in 2020.
GLAD height	Tree canopy height layer estimated from Landsat and GEDI data^10^, used to create a mask of minimal natural and planted forest height.
GFM-FT 2020	Global Forest Management – Forest Types (GFM-FT) map is trained on GFM 2020 training data (data by courtesy of Dr. M. Lesiv and Dr. S. Fritz, IIASA), which is an update to^16^. The classes were reassigned to the forest types as used in this work (natural forest, planted forest, tree crops, other). The data is used as an additional mask for natural forest (probability of GFM-FT natural forest class >0.5), and non-natural forest land (probability of GFM-FT natural forest class <0.3). We also threshold it based on the Copernicus Global Land Cover^73^ tree coverage layer, as originally done in^16^.
Drivers	Drivers of forest loss between 2000 and 2020 at 1 km resolution^6^. The classes are: (1) permanent agriculture, (2) hard commodities, (3) shifting cultivation, (4) logging, (5) wildfire, (6) settlements and infrastructure, and (7) other natural disturbances. For this work, we first combined the drivers data with GLAD GFC tree cover and forest loss year layer^9^, to only keep areas which had tree cover >10% in 2020, and which experienced forest loss between 2001 and 2020. After this combination, the resulting drivers data has a 30 m resolution matching the GLAD GFC data. We used this data as an additional mask for potentially natural forest after wildfires, and for non-natural forest land after likely permanent conversion following a deforestation event (permanent agriculture, hard commodity, and settlements and infrastructure classes).

The overall process for natural forest class assignment consisted of the following steps (see Fig. 3 for details):

1. We created the initial natural forest class as an overlapping combination of sources: natural forest equivalent classes from TMF, SBTN, GFT2020, GFM-FT (p(natural) >0.5), as well as PHTF, European and Canadian primary forests, US mature old-growth, and boreal primary and old secondary forests. We also included areas of forest loss caused by wildfires, assuming natural regrowth.
2. From these initial natural forest annotations, we removed areas that experienced recent permanent forest cover loss or deforestation (2000–2020), and areas likely non-natural according to GFM-FT (p(natural) < 0.3).
3. We applied a forest mask, limiting the forest area to locations with tree heights greater than 5 m^10^, or locations that experienced natural disturbance between 2000 to 2020^6^, or locations characterized as forest in JRC Forest Types^19^.
4. After constructing the planted forest and tree crops classes (see Fig. 3), we masked out any ambiguous pixels that overlapped with these classes and denoted them as unknown.

We constructed the supplementary classes similarly using a reduced number of sources, as outlined in Fig. 3. We also applied the forest mask to the planted forest class since it is expected to conform to the forest definition. We applied the inverse of the forest mask to the other vegetation, built, water, ice/snow and bare classes. For the ‘other vegetation’ class, which can be ambiguous with tree classes, we adopted a more conservative approach, assigning that label only if all relevant label sources agree (including SBTN, WorldCover, and indicating no forest in GFC tree cover and in our forest mask).

**Fig. 4:**
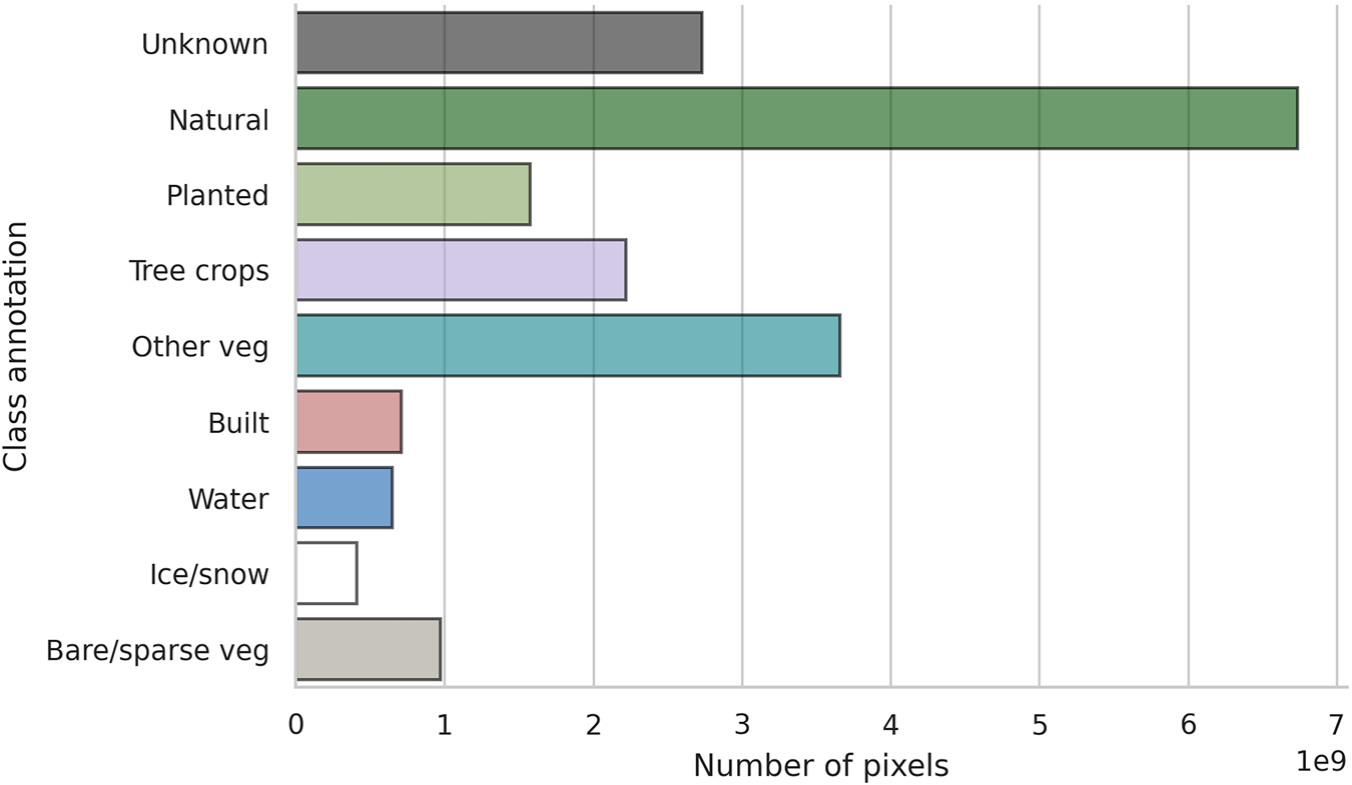
Class distribution at pixel level in the training data. The x-axis denotes the number of pixels in billions (10^9^).

**Fig. 5:**
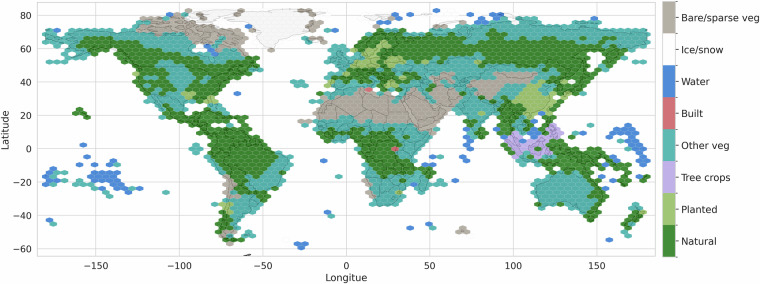
Global spatial distribution of training data. Each hexagon denotes the dominant class within its area.

The final distribution of determined class annotations per 10 m pixels in the training data is reported in Fig. 4. The natural forest class, the most important one, covered 34.3% of the training data pixels. Hard negatives (planted forest, tree crops and other vegetation) also covered a significant area with 37.9%. 13.9% of pixels were denoted as unknown due to unavailable or inconclusive/ambiguous sources. The global spatial extent of the training data is shown in Fig. 5, where only the local majority class is denoted.

### Model inputs

For each sample location, we constructed a model training example of predictor variables by combining multi-temporal multi-spectral data from Sentinel-2, elevation and topology data from FABDEM^27^, and the geographic location of the sample.

We used multi-spectral imagery from Sentinel-2 surface reflectance data (Level-2A), originally processed by sen2cor^28^. We masked out cloudy areas using Cloud Score+ with the default clear threshold of 60%^29^. We utilized 10 Sentinel-2 bands that are sensitive to land cover (B2, B3, B4, B5, B6, B7, B8, B8A, B11, B12), resampling all to 10 m resolution. During dataset generation, we aggregated all temporal cloud-free Sentinel-2 images for 2020 into four three-months seasonal composites (December-February, March-May, June-August, September-November, corresponding to winter, spring, summer, autumn in the Northern Hemisphere) using a median temporal filter. This resulted in four 10-band images per sample, giving final dimensions for Sentinel-2 inputs of (4, 128, 128, 10) representing (temporal dimension, height, width, number of frequency channels).

We obtained elevation data from the Copernicus GLO-30 Digital Elevation Model^30^, based on interferometric synthetic aperture radar (InSAR) data acquired by the TanDEM-X mission between 2011 and 2015. We used the FABDEM variant that additionally removed estimated forest and building heights^27^. In addition to the surface elevation above sea level, we computed the local slope and the aspect angle of the slope. After resampling the original 30 m data to 10 m resolution, the input dimensions were (1, 128, 128, 3), with the 3 bands representing elevation, slope, and aspect.

For global context information, we included the geographical location (latitude and longitude at the center of each sample) represented as unit-sphere Cartesian coordinates.

**Fig. 6:**
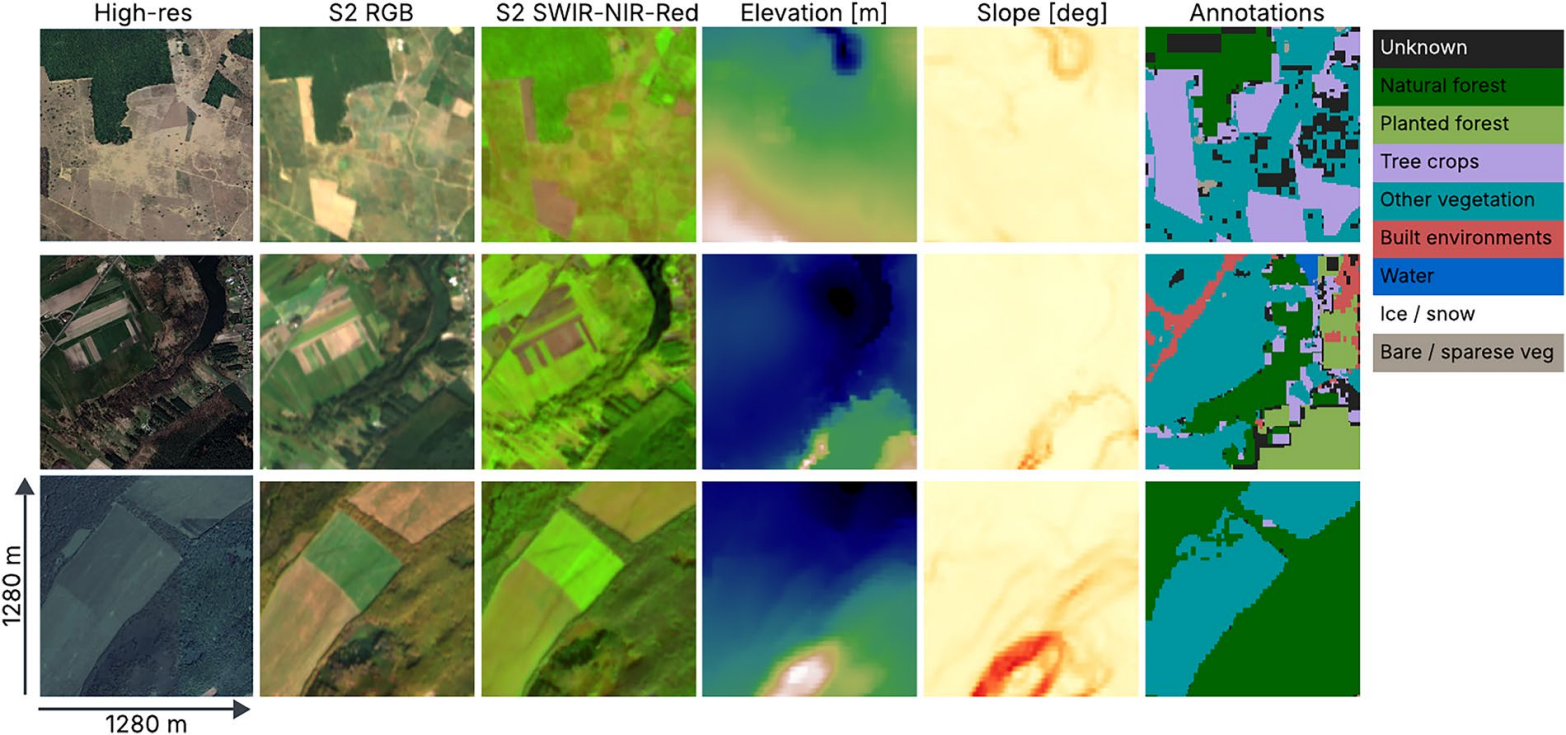
Examples of three training locations shown in very high resolution satellite imagery from Google Maps, with model input examples from left to right: (2) Sentinel-2 Red-Green-Blue bands, (3) Sentinel-2 SWIR-NIR-Red bands, (4) elevation, (5) slope, and (6) class annotations. To the right is the color map for the class annotations.

Fig. 6 shows examples of model input data, including multi-spectral composites of Sentinel-2 data, elevation data, and the constructed label mask that the model is trained to predict.

### Model training

**Fig. 7:**
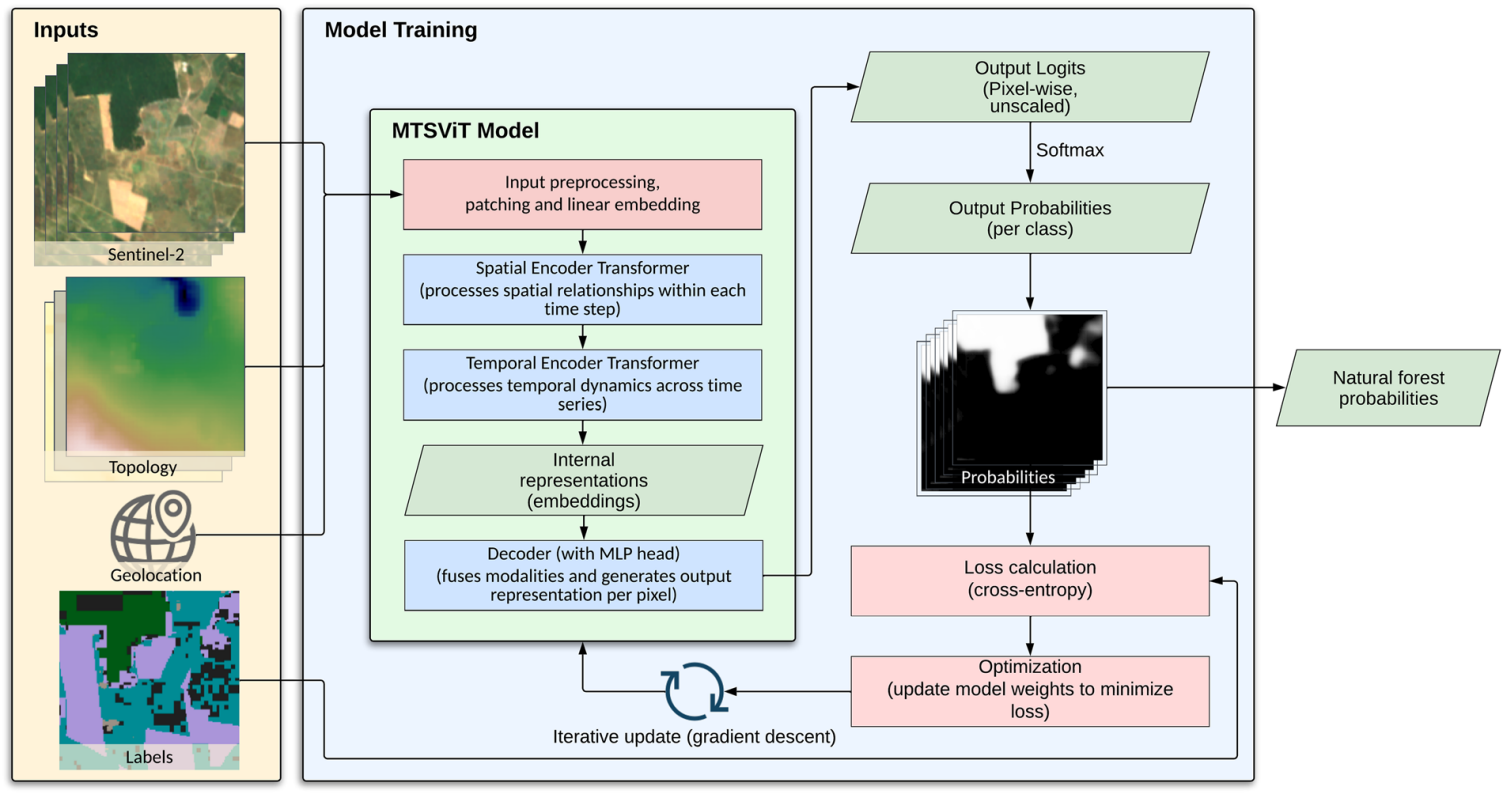
An overview of model training and the multi-modal spatio-temporal vision transformer (MTSViT) model. The model takes Sentinel-2 time-series imagery and topography data as inputs, processes each data source independently into patch embeddings, and passes them through shared spatial and temporal encoders to produce spatio-temporal embeddings. The embeddings from both modalities are then fused in a multi-modal decoder and passed through a segmentation head to estimate the class probabilities per pixel. During training, the weights of the model are iteratively updated to minimize the loss objective (cross-entropy between these probabilities and the labels).

Our approach utilized a novel Multi-modal Temporal-Spatial Vision Transformer (MTSViT) model (Fig. 7), an adaptation of the Vision Transformer (ViT) architecture^31,32^, engineered to effectively process multi-modal time-series satellite data as input. The ViT model adapts the Transformer architecture, originally designed for natural language processing, to image recognition by treating an image as a sequence of smaller image patches.

In our MTSViT, we initially divided each input image into 8 × 8 pixel patches, resulting in (128/8)^2^ = 256 spatial patches per image. We then projected each 8 × 8 pixel patch into a 192-dimensional vector representation (a ‘token’) via a learned linear embedding. This process converts each input image into a sequence of 256 such tokens, which is the standard input format for a Vision Transformer model. Subsequently, a two-stage encoding process extracted both spatial and temporal information. First, a spatial transformer encoder operated on these tokens (independently for each data source and time step) using multiple transformer layers (*depth* = 2) with self-attention^33^. This stage captured spatial relationships within each image at each time point. Second, a temporal encoder (*depth* = 2) processed the output of the spatial encoder to extract temporal dynamics across the time series (again independently for each data source and spatial token). Following this encoding, we fed the compressed spatial and temporal information into a transformer decoder (*depth* = 4). The decoder’s output was then processed by a multi-layer perceptron (MLP, with hidden layer *dimension* = 768) to predict the spatial maps of interest (pixel-wise class logits). We converted the model’s direct outputs (logits, unscaled class log-probabilities) to normalized probabilities using a softmax operation^34^.

The model’s architecture is designed to leverage the distinct information content of each input modality. The spatial encoder processes the 8 × 8 pixel patches within each seasonal composite, allowing it to learn textural and fine-grained spatial patterns indicative of different land cover types (e.g., the regular patterns of plantations versus the heterogeneous texture of natural forests). The temporal encoder then processes the sequence of these spatial representations across the four seasons, enabling it to capture the unique phenological signatures of different vegetation types. Finally, the decoder fuses these spatio-temporal embeddings with the topographic data (elevation, slope, aspect) and geographic location, enabling the model to learn complex relationships between land cover, terrain, and biome-specific characteristics.

Both the encoder and decoder transformer components of our MTSViT were lightweight, consisting of a small number of transformer layers (2 and 4, respectively) with 6 attention heads each. This design effectively captured spatial, temporal, and multi-modal interactions without excessive computational cost. The specific architectural parameters were: embedding size = 192, number of attention heads = 6, temporal patch size = 1, spatial patch size = 8, and MLP dimension = 768. We found that ensembling five MTSViT models with different random initializations improved performance, with the final prediction generated by averaging their softmax probability outputs.

We trained the model weights by minimizing the cross-entropy loss function using gradient descent with the Adam optimizer^35^ on minibatches of size 512^34^. During model exploration, we trained models for 10 epochs on the *train* split of the data and evaluated them on the *test* split (10% of land patches of size 100  × 100 km^2^ randomly distributed and not overlapping with the *train* split). During each training iteration, we applied random data augmentations (synchronous rotations and flipping) to the input data. We trained the model on 64 TPUv3 accelerator chips. We used a standard Adam optimizer with learning rate = 0.001, weight decay = 3e-5, and a cosine learning rate decay schedule with a warmup of 10% of the training duration. We also applied gradient clipping (threshold value = 1.0) to stabilize training and to prevent the gradients from becoming too large. Note that we ignored pixels with the class *unknown* during training (they did not contribute to the loss); the model therefore never learned to predict that class but still estimated the likelihood of other classes for pixels labeled as unknown. We performed hyperparameter tuning on model configuration, input data sources, and label construction. We evaluated the model on F1-score (a harmonic mean of the user’s and producer’s accuracies) and overall accuracy metrics on the *test* dataset split.

We found that ensembling five MTSViT models with different random initializations improved performance. Once we determined the best model inputs and model and training configuration, we retrained an ensemble of five models on the combined *train* and *test* splits for final map generation. The final probability for each class was calculated by averaging the softmax probabilities from the five individual models in the ensemble. A completely independent validation dataset, which was never seen during training, was used for the final map evaluation in the Technical Validation section.

While a single model architecture is used globally for consistency, its design allows it to learn regionally-specific patterns. The inclusion of geographic coordinates provides the model with explicit location context, while the multi-temporal Sentinel-2 composites enable it to learn the distinct phenological signatures of different biomes (e.g., strong seasonality in boreal forests vs. evergreen behavior in tropical rainforests). In this way, the model learns a globally consistent but locally sensitive mapping function.

### Map construction

After the model is trained we created an inference dataset covering all land areas between −65 and +84 degrees latitude for final map construction. We then used the final trained model ensemble to estimate the probability for the *Natural* forest class for each inference sample. To reduce tiling and patching artifacts, we performed inference using overlapping samples, with a distance between inference sample centers of 210 m (the height and width of each sample is 1280 m). While non-overlapping samples were used during training, this overlapping inference strategy was employed to produce a smooth, seamless final map. We weight-averaged the predictions for overlapping pixels based on the inverse Euclidean distance of the pixel to its respective sample center.

### Model uncertainty and calibration assessment

Predictions from neural network models inherently possess uncertainty. The two primary sources^36^ are: epistemic uncertainty (related to model parameters) and aleatoric uncertainty (related to inherent input data ambiguity). For our binary classification task (natural forest vs. other), the predicted natural forest probability serves as an approximate measure of model confidence, albeit with certain limitations. It is well-established that class probabilities generated by deep learning models can be miscalibrated, often exhibiting a tendency towards overconfident predictions (probabilities clustering near 0 or 1)^37,38^.

To enhance the reliability of our probability estimates, we implemented several strategies. First, we used an ensemble of 5 independently trained models to mitigate epistemic uncertainty. Second, we evaluated the calibration of our final probability estimates using an independent validation split derived from GFM^16^, updated to 2020 (see Technical Validation section), which was never seen during training. Specifically, we assessed whether our predicted forest probabilities aligned with the actual observed forest proportions in this hold-out dataset using adaptive histogram binning^39^.

Our calibration analysis revealed instances of overconfidence in certain probability ranges. Consequently, we applied temperature scaling^40^ with a temperature parameter *T* = 1.4 to recalibrate the model’s output probabilities. Note that this calibration rescaled the probabilities but did not affect the evaluation metrics in the Technical Validation section at the optimal probability threshold. After probability calibration, the generated map represents the estimated probabilities of the natural forest class at 10 m resolution.

We quantized the final map probabilities into 0.4% intervals to reduce file size.

## Data Records

The natural forest probability map is available for download at (10.25452/figshare.plus.28881731)^41^, and on the Google Earth Engine (GEE) (https://developers.google.com/earth-engine/datasets/catalog/projects_nature-trace_assets_forest_typology_natural_forest_2020_v1_0_collection). A GEE App to analyze the data is available at (https://nature-trace.projects.earthengine.app/view/natural-forests-2020). The dataset is licensed under the Creative Commons Attribution 4.0 International License (CC BY 4.0). We provide the dataset as Cloud Optimized GeoTIFFs (COGs). The map uses the Universal Transverse Mercator (UTM) coordinate system, has a spatial resolution of 10 m per pixel, and contains unsigned 8-bit integer values (0-250) representing quantized probability values. Each UTM zone is split into 100 smaller tiles/files, resulting in 37,166 files containing land cover.

To reduce disk space and enable faster loading, we quantized the probability values into the integer range of 0 to 250 (stored as unsigned 8-bit integers). To retrieve the estimated probabilities, users need to convert the integer values to floats and divide by 250. This quantization implies that the map’s probability resolution is 0.4%.

The probabilities can be used to create a binary natural forest map by setting a probability threshold (either the recommended value of 0.52, or another threshold that is estimated for a particular research objective in a specific region of interest). Fig. 1 shows the estimated global extent of the natural forests using the 0.52 probability threshold.

The tabular validation data that was used for accuracy assessment is available at (10.25452/figshare.plus.30051517)^42^. It is licensed under the Creative Commons Attribution 4.0 International License (CC BY 4.0). This dataset is in a comma-separated values (CSV) file, consisting of 2,072 records with sample locations, natural forest class label, and the strata index.

## Technical Validation

### Accuracy assessment and comparison with other datasets

We performed evaluation and validation of our map based on the Global Forest Management (GFM) validation dataset^16^, which we updated to 2020 for this study. This validation dataset has no intersection with GFM-FT training data used during model training. We performed statistically rigorous accuracy assessment, adjusting for the different strata following established methods^43,44^.

We updated the GFM validation dataset for 2020 by visually re-assessing and re-labeling validation plots from the GFM 2015 validation dataset from^16^ that might have experienced natural forest changes between 2015–2020. We simplified the labeling task to assigning one of two labels: *natural forest* (class 1, corresponding to original GFM classes 11 (naturally regenerating forests without signs of management) and 20 (naturally regenerating forests with signs of management)) versus *other* (class 0, all other GFM classes). To determine which plots potentially experienced changes, we assessed Global Forest Change^9^ data between 2015 and 2020. This resulted in a subset of 56 plots (out of 816 total validation plots originally labeled as natural forest in 2015) that showed some tree cover loss. We did not assess other classes under the assumption that a transition from non-natural forest to natural forest was highly unlikely over this period. Two to three experts visually re-assessed each of these 56 plots using the latest satellite imagery (very high-resolution imagery in Google Earth Pro and ESRI World Imagery Wayback, and various contextual layers in Google Earth Engine) and re-assigned labels for 2020 where necessary.

It is important to note that this dataset was originally collected using a stratified random sampling design^16^. However, our current analysis focuses on a binary classification of *natural forest* versus *other*. The full dataset contains 2,072 sample plots globally, which for our binary assessment correspond to 800 plots of *natural forest* and 1,272 plots of *other*. Due to this difference in classification schemes, the original strata defined in^16^ do not directly correspond to our map classes. Therefore, we employed *general estimators for stratified random sampling* as described in^43^ to ensure statistically rigorous accuracy and area estimation. This approach accounts for the varying inclusion probabilities associated with the original strata. The accuracy assessment produced estimates of accuracies that acknowledged the complexities arising from the differing stratification.

Since the GFM data provided a label for a 100  × 100 m plot, while our map and others have predictions at 10 to 30 m pixels, we developed the following approach to accurately evaluate against this dataset without bias. We assumed that GFM labels correspond to  >50% area cover within the 100  × 100 m plots. For probability maps, we first thresholded all pixels within the 100 m area using a selected probability threshold. Then, we assigned the plot-level prediction to the *Natural forest* class based on the majority (>50%) of pixel predictions within the plot. We applied the same procedure to other evaluated datasets for consistency. Because the validation sampling unit size was 100  × 100 m, we did not assess the accuracy of spatial details at finer resolution (e.g., 10 m).

**Fig. 8:**
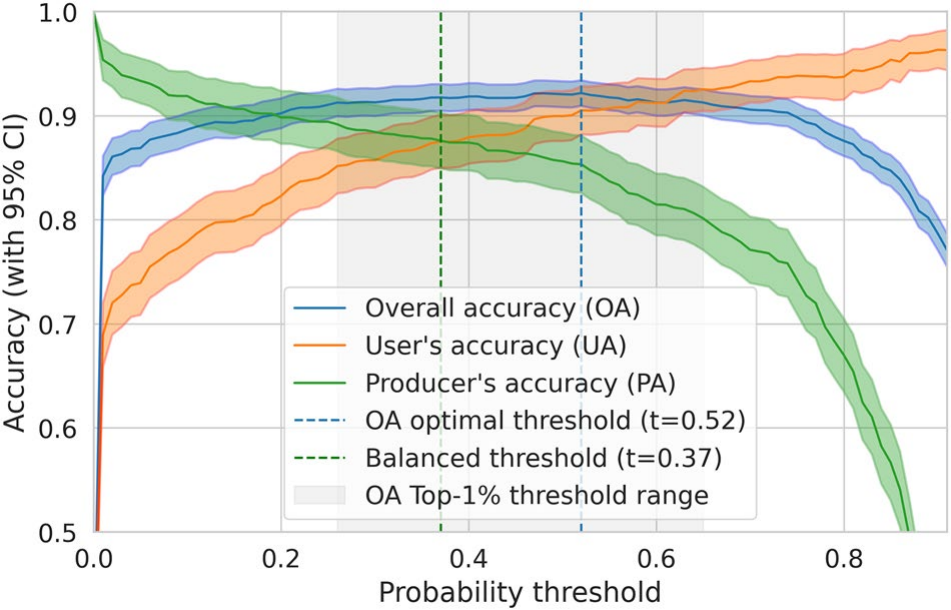
User’s accuracy, producer’s accuracy, and overall accuracy on the Global Forest Management (GFM) 2015 validation data^16^ updated to 2020. The shaded areas include 95% confidence intervals. Also denoted are the optimal OA and balanced probability thresholds, as well as the range of probabilities within 1% of maximal OA.

Selection of the probability threshold is an important step and can be adjusted for particular use cases, depending on whether user’s or producer’s accuracy (UA or PA) should be prioritized, and based on map quality in a particular region. Fig. 8 shows the overall accuracy (OA), UA, and PA, plotted against the probability threshold. The graph also shows the 95% confidence intervals computed as  ±1.96*SE (standard error) of the metrics. The behavior of the User’s Accuracy (UA) curve at low thresholds is a result of the stratified sampling design of the validation dataset. The UA is calculated as the ratio of correctly classified positive samples to all samples classified as positive, area weighted by the strata. At a threshold of 0, all samples are classified as positive, so the UA is simply the proportion of positive samples in the validation set, area weighted by the strata.

The vertical bars in Fig. 8 denote specific probability thresholds. The probability threshold with the highest OA is 0.52. However, as observed for the optimal overall accuracy, this threshold yields high user’s accuracy, but lower producer’s accuracy, representing a trade-off that reduces commission errors at the cost of more omission errors. Alternatively, one could choose a balanced threshold at 0.37, where UA is similar to PA, with only a minor drop in OA compared to the maximum. At this threshold the commission and omission errors are balanced on the GFM 2020 validation dataset. Note also that OA is not very sensitive to a wide range of probabilities, and the greyed area denotes the range where OA is within 1% of the top OA.

For comparison, we also evaluated other recently released natural forest cover maps:

1. **GFT2020**: Joint Research Center’s (JRC’s) Forest Type map^19^. We combined classes 1 (naturally regenerating forest) and 10 (primary forest) to represent natural forest.
2. **UMD IPCC**: University of Maryland’s forest map for the Intergovernmental Panel on Climate Change (IPCC) assessment^22^. We constructed the natural forest class by combining all 3 relevant classes (primary and young and old secondary forests).
3. **SBTN v1.1**: Science Based Targets Network map denoting natural lands, including forests^21^. We constructed the natural forest class by combining classes 2 (natural forests), 5 (natural mangroves), 8 (wet natural forests), and 9 (natural peat forests)^21^.
4. **Forest Persistence v0**: Forest Data Partnership’s (FDaP’s) undisturbed forest score (0 to 1) at 30 m resolution, for 2020^20^.

The evaluation results using a stratified estimator (combined ratio estimator)^43,44^ on the updated GFM 2020 validation data are shown in Table 4. We report the results at the overall accuracy optimal probability or confidence score threshold *t*_*oa*_, which was 0.52 for our map (NFW) and 0.57 for Forest Persistence map. Alongside the accuracy metrics, we report the estimated standard error in the parentheses. We found that the overall accuracy of the NFW map was 92.2% (±0.6%), which was 3 percentage points higher than the next best map in this comparison.

**Table 4:** Evaluation results using a stratified estimator on Global Forest Management (GFM) 2015 validation data^16^ updated to 2020 for this study.

Map	Overall acc. (SE)	User’s acc. (SE)	Producer’s acc. (SE)
GFT2020	89.2 (0.7)	85.2 (1.4)	81.5 (1.5)
UMD IPCC	85.4 (0.8)	88.1 (1.4)	64.7 (1.8)
SBTN v1.1	86.0 (0.8)	84.8 (1.5)	70.4 (1.8)
Forest Persistence (*t*_*oa*_ = 0.57)	88.7 (0.7)	81.0 (1.2)	86.2 (1.4)
ForestPersistence (*t*_*balanced*_ = 0.62)	88.3 (0.7)	82.3 (1.2)	82.5 (1.6)
Our map (*t*_*oa*_ = 0.52)	92.2 (0.6)	90.5 (1.2)	85.3 (1.4)
Our map (*t*_*balanced*_ = 0.37)	91.7 (0.7)	87.5 (1.3)	87.6 (1.4)

Standard error (SE) of the accuracy metrics is reported in the parentheses.

Table 5 presents the evaluation results per continent for our map, using the same globally optimal probability threshold (*t*_*oa*_ = 0.52). Although we used the global threshold, we also observed that the locally optimal threshold could vary by continent. The map performs best in North and South America as well as in Asia, with lower overall accuracy in Europe, Africa and Australia/Oceania.

**Table 5:** Evaluation per continent (at global optimal OA threshold).

Continent	Overall acc. (SE)	User’s acc. (SE)	Producer’s acc. (SE)
Africa (*t* = 0.52)	89.0 (1.7)	92.9 (2.2)	70.1 (4.6)
Asia (*t* = 0.52)	94.0 (0.9)	91.8 (1.9)	88.3 (2.0)
Australia and Oceania (*t* = 0.52)	86.3 (4.3)	93.0 (6.1)	53.0 (11.6)
Europe (*t* = 0.52)	89.2 (2.1)	82.5 (3.4)	82.3 (5.1)
North America (*t* = 0.52)	93.5 (1.4)	87.1 (2.9)	92.9 (2.8)
South America (*t* = 0.52)	94.7 (1.6)	95.5 (2.4)	94.4 (1.8)

Standard error (SE) of the accuracy metrics is reported in the parentheses.

### Error analysis

**Fig. 9:**
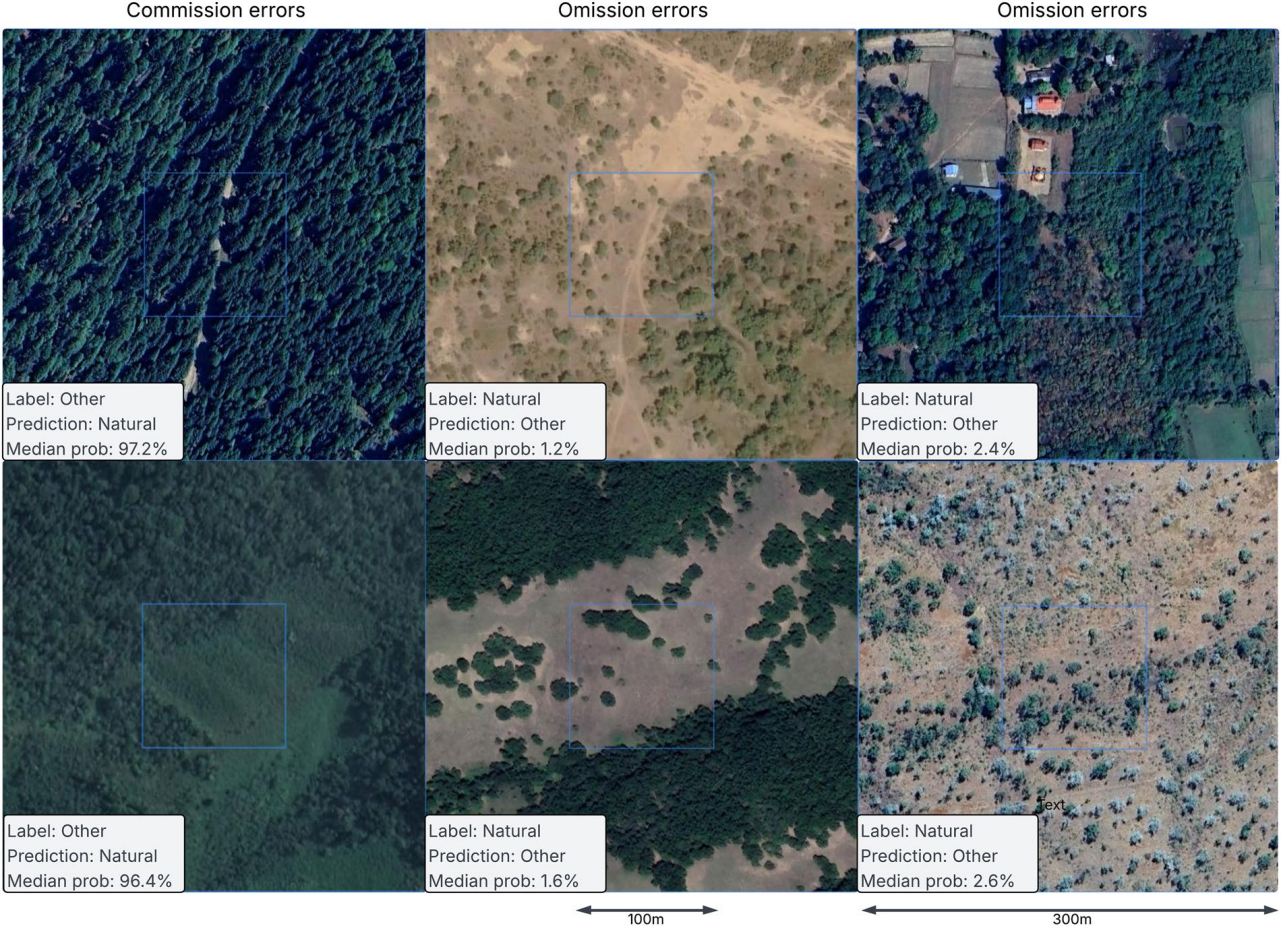
Examples of high-confidence commission and omission errors. The central square of each example covers the 100  × 100 m area that is being evaluated. On the left: commission errors, potentially misinterpreting planted forest as natural forest. In the center and to the right: omission errors in sparse trees areas and close to human settlements and agriculture.

At very high probability thresholds, there are fewer samples where the map confidently predicts natural forest. The few error outliers disproportionately strongly affect UA. At a probability threshold of 0.95, only 47 validation samples were predicted as natural forest, 4 of which had the reference label *other* (resulting in a commission error rate of 8.5% for this high threshold). We analyzed several high-confidence commission errors and observed quite ambiguous and difficult cases. Fig. 9 demonstrates some high-confidence examples of apparent errors. The first two examples on the left show commission errors where the map predicted natural forests, while the reference label indicated potentially planted forest according to^16^.

Conversely, at a probability threshold below 0.05, there were 60 omission errors where the map confidently predicted *other*, but the reference label was *natural forest* (out of 997 samples predicted as *other* with *p* < 0.05; representing a 6% omission error rate among these high confidence *other* predictions). Often we observed that the model did not predict natural forest if the trees were very sparse or close to settlements with agriculture, as shown on the right examples in Fig. 9.

### Limitations

While this study provides a novel global baseline map of natural forests for 2020, it is important to acknowledge certain limitations in our map (assessed at the OA optimal probability threshold of 0.52):

- Agroforestry and smallholder systems: Some complex agroforestry systems (e.g., with shaded tree crops, such as shaded cocoa plantations in West Africa) and smallholder agricultural mosaics can be difficult to distinguish from natural forest using satellite data alone. The misclassification is particularly apparent in some areas in Southeast Asia and Latin America.
- Planted and orchards vs. natural forest differentiation: Distinguishing planted forests from naturally regenerating forests can be challenging using only remote sensing satellite data. This is especially prevalent in regions like the boreal zone, where some natural forests have lower species diversity and planted forests are harvested with longer rotation times (up to 100 years) compared to the tropics^45^. These long rotations and homogeneous stands can mimic the characteristics of natural or old-growth forests, making them difficult to separate based on spectral and textural features alone. Consequently, our map (with a probability threshold of 0.52) tends to overestimate natural forest in Scandinavia. We observed similar overestimation in some parts of temperate forests in the United States Northwest and Midwest. Similarly we observed some orchards (for example in northern Turkey) to be misclassified as natural forest.
- Sparse natural forest, such as savanna, are often at the threshold of natural forest definition for the tree canopy height and coverage ratios. It is not easily possible to determine the correctness or errors of the map predictions.
- Post-disturbance ambiguity: Forest type assignment immediately after a disturbance event (e.g., fire, logging) is inherently ambiguous. It may not be clear from satellite imagery whether the forest will regenerate naturally or if the land will be converted to another use (e.g., plantation, agriculture).
- Other ambiguities: Areas of potential confusion could include large parks within urban areas, or planted tree belts that meet forest definition criteria but are not natural.
- Input data quality: The accuracy of our natural forest map is intrinsically linked to the quality and consistency of the various input datasets used for training label generation (Tables 2, 3). These datasets were created using different methodologies, spatial resolutions, temporal ranges, and definitions. Some label layers were the outputs of other models, and are therefore limited by the quality of those models. While our approach aimed to harmonize sources and mitigate the impact of individual dataset errors, inconsistencies and inaccuracies in the underlying data could still influence the final map.

An important avenue for improvement will be to address these limitations in future versions of the dataset.

## Usage Notes

Except for the probability quantization and calibration, we released the map without any additional post-processing. Consequently, users may choose to apply post-processing heuristics to optimize the map for specific use cases. For example, users might want to refine the natural forest extent by filtering out areas using a minimal tree canopy height threshold. There are various regional and global tree canopy height maps available (e.g.^10,46–48^) that could be used for this task.

After probability threshold selection and creating a binary natural forest map, users may also choose to remove predicted natural forest patches with areas smaller than a specific threshold (e.g. 0.5 hectares according to FAO).

### Tiling artifacts

The model used a spatial context window of 1280 m when making predictions. While our overlapping inference approach aimed to minimize discontinuities between adjacent prediction windows, subtle tiling artifacts might still appear in the probability map when merging neighboring prediction windows, particularly near the corners of the underlying inference tiles. These artifacts usually disappear or become negligible after applying a probability threshold to create a binary map.

### Probability threshold selection

Choosing an optimal probability threshold is crucial for balancing different types of errors when creating a binary classification map from the probability layer, and this decision is inherently tied to the specific application and the desired error characteristics. For a given application and desired balance between commission (false positives) and omission (false negatives) errors, users should select the probability threshold by analyzing the trade-off between User’s Accuracy (UA) and Producer’s Accuracy (PA).

The plot in Fig. 8 can guide threshold selection based on global validation data. Based on our global analysis, we recommend using the threshold between 0.3 to 0.55, depending on the desired balance between UA and PA. However, if local evaluation data are available, we recommend using a data-driven approach: recompute the accuracy metrics for the region of interest across different thresholds and select the threshold best suited to the local context and application needs.

Some general guidance for probability threshold selection:

- To prioritize User’s Accuracy (minimizing commission errors/false positives, i.e., high confidence that mapped forests are truly forests), select a higher threshold from the curve in Fig. 8 where UA is high.
- To prioritize Producer’s Accuracy (minimizing omission errors/false negatives, i.e., capturing most of the actual forest), select a lower threshold where PA is high.
- To seek a balance, choose a threshold near the intersection point of the UA and PA curves in Fig. 8, or where both accuracies are acceptably high.

## Data Availability

The natural forests of the world 2020 dataset is available at Figshare under the following link: 10.25452/figshare.plus.28881731^41^.

## Code availability

We generated the training dataset and the final map using the *GeeFlow* library (https://github.com/google-deepmind/geeflow) that uses Google Earth Engine^49^ as the backbone. The code for model training, inference, and evaluation is available in the *JEO* code repository (https://github.com/google-deepmind/jeo).
